# Current practice of intracranial pressure monitoring in children with severe traumatic brain injury—a nationwide prospective surveillance study in Germany

**DOI:** 10.3389/fped.2024.1355771

**Published:** 2024-02-09

**Authors:** Pia Brensing, Sandra Greve, Rayan Hojeij, Philipp Dammann, Ursula Felderhoff-Müser, Christian Dohna-Schwake, Nora Bruns

**Affiliations:** ^1^ Department of Pediatrics I, Neonatology, Pediatric Intensive Care Medicine, and Pediatric Neurology, University Hospital Essen, University of Duisburg-Essen, Essen, Germany; ^2^ C-TNBS, Centre for Translational Neuro- and Behavioral Sciences, University Hospital Essen, University of Duisburg-Essen, Essen, Germany; ^3^ Department of Neurosurgery and Spine Surgery, University Hospital Essen, University of Duisburg-Essen, Essen, Germany

**Keywords:** children, severe traumatic brain injury, intracranial pressure monitoring, outcomes, ICP, neuromonitoring

## Abstract

**Background:**

For management of severe traumatic brain injuries (sTBI) in children, the overall level of evidence to guide diagnostic and therapeutic procedures is low. Since 2016, international guidelines have subsequently suggested invasive intracranial pressure (ICP) monitoring in patients with initial Glasgow Coma Scale (GCS) ≤8. In Germany, ICP monitoring was an individual case decision from 2011 until the 2022 update of the German pediatric TBI guideline. The aim of this study was to evaluate current clinical practice of invasive ICP monitoring in Germany in children <10 years with respect to guideline recommendations.

**Methods:**

Anonymized clinical data on sTBI cases <10 years of age were collected in a nationwide prospective surveillance study via the German Pediatric Surveillance Unit ESPED from July 2019 until June 2022. Inclusion criteria for the surveillance study were sTBI (initial GCS ≤8) or neurosurgery following TBI. For this analysis, only cases with GCS ≤8 were subject to the present analysis. Descriptive analyses were performed to assess the proportion of ICP monitored patients and describe the cohort.

**Results:**

Out of 217 reported cases, 102 cases met the inclusion criteria and thus qualified for ICP monitoring. Of these, 37 (36%) received ICP monitoring. Monitored patients were older, had lower median GCS values at presentation (4 vs. 5), higher mortality (32% vs. 22%), and were more frequently diagnosed with cerebral edema (68% vs. 37%).

**Conclusion:**

In children <10 years with sTBI, the present clinical management regarding ICP monitoring deviates from the current German national and international guidelines. The reasons remain unclear, with the low level of evidence in the field of ICP monitoring and the recency of changes in guideline recommendations as potential contributors. Prospective interventional studies should elucidate the benefit of ICP monitoring and ICP directed therapies to provide evidence-based recommendations on ICP monitoring.

## 1 Introduction

Traumatic brain injury is a major contributor to mortality and acquired morbidity in children across the world (1). In Germany, 458,844 children <18 years were hospitalized with TBI between 2014 and 2018, resulting in an incidence rate of 687/100,000 child years (CY) and mortality of 0.67/100,000 CY (0.1%) (2). Age <2 years, GCS <6, hypothermia, hyperglycemia, and coagulation disorders are associated with increased mortality (3). Due to the brain's low tolerance towards hypoxia, optimizing the timeframe between insult, diagnostics and targeted neuroprotective measures is particularly crucial to reduce long-term sequelae and mortality.

**Figure 1:**
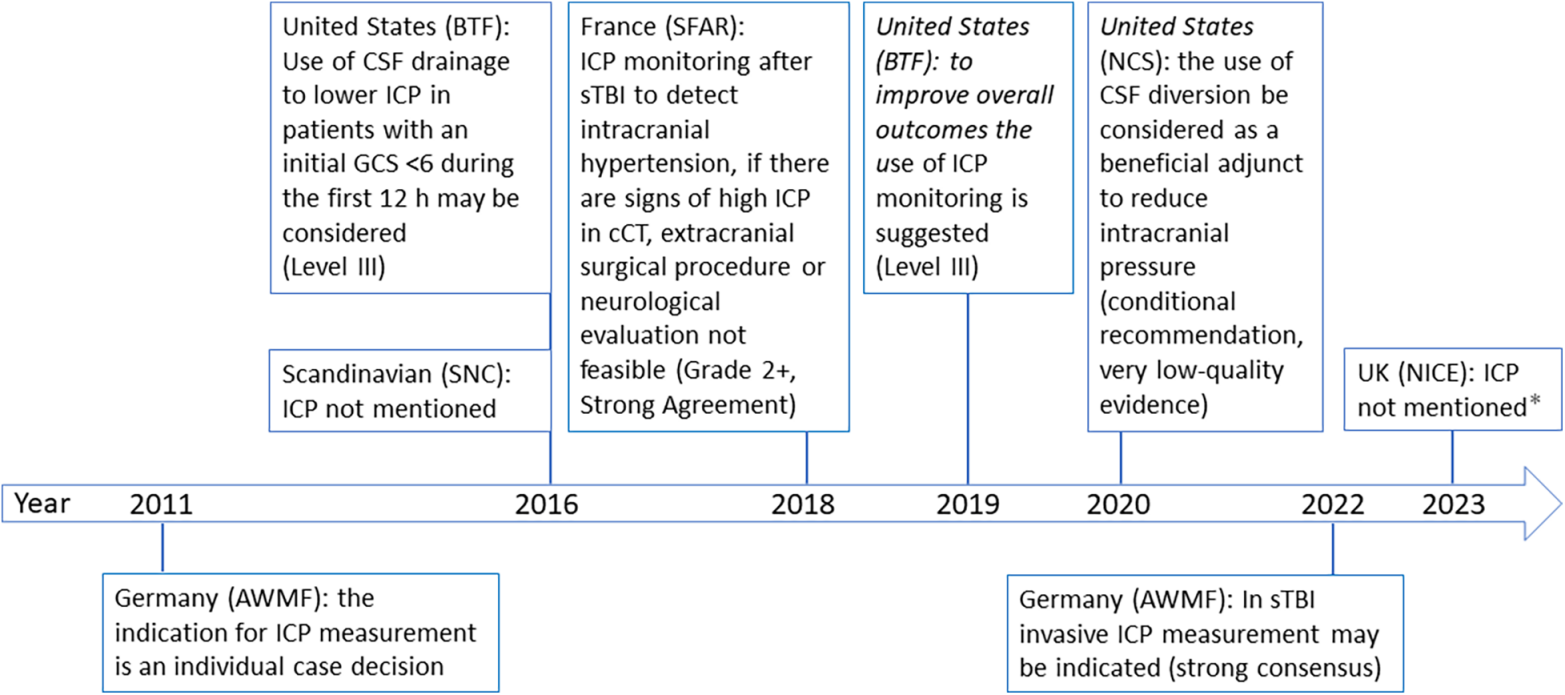
Timeline of international guideline recommendations. AWMF, working group of scientific medical societies (4, 8); BTF, Brain Trauma Foundation (6, 22); cCT, cranial computertomography; GCS, glascow-come-scale; ICP, intracranial pressure; NCS, Neurocritical Care Society (23); NICE, the National Institute for Health and Care Excellence (24); SFAR, French Society of Anesthesia and Intensive Care Medicine (7); SNC, Scandinavian Neurotrauma Committee (25). * guideline dedicated to give recommendations on triage in the emergency department but not on ICP monitoring.

Adherence to specific therapy recommendations has reduced mortality and improved functional outcome after severe traumatic brain injury (sTBI) (4). Immediate protective intubation, normalization of vital parameters (5), and basic neuroprotective measures are the cornerstones of sTBI management (6). Additionally, invasive intracranial pressure (ICP) monitoring is suggested for comatose [Glasgow Coma Scale (GCS) value ≤8] or continuously sedated patients by international guidelines and in the 2022 update of the German national guideline with strong consensus but low level of evidence (4, 6, 7). The previous German guideline, which was in effect since 2011 and expired in 2017, acknowledged the potential benefits of ICP monitoring but declared it as individual case decision (8) (Figure 1).

In spite of the tentative national guideline recommendation, a survey in 2017 among 29 German pediatric intensive care units (PICU) found that 25 (86%) units declared to use ICP monitoring always or often in patients with severe TBI (9). Our study aimed to verify how many children <10 years with sTBI who fulfilled criteria for ICP measurement in fact received this treatment and describe the characteristics of these patients via a prospective nationwide surveillance study in Germany.

## 2 Methods

### 2.1 Data collection

This prospective nationwide surveillance study collected data on sTBI in children <10 years via the German Pediatric Surveillance Unit (ESPED) from July 1st, 2019 until June 30th, 2022. Inclusion criteria were age <10 years, initial GCS ≤8 or intracranial injury requiring neurosurgery.

ESPED is the official German Pediatric Surveillance Unit of the German Society of Pediatric and Adolescent Medicine (DGKJ) and provides the infrastructure to conduct nationwide surveillance studies (www.unimedizin-mainz.de/esped). In 2022, 346 of 381 (90.8%) German children's hospitals contributed to the ESPED network (10). ESPED actively surveys newly diagnosed children with specific rare diseases via monthly requests to pediatric hospitals. If a new case is reported, a case report form including anonymized basic demographic data, clinical and laboratory characteristics, and medical history has to be completed. From July 1st, 2019 until December 31st, 2020, data were collected via paper-based questionnaires. From January 1st, 2021 onwards, data were collected via a web-based application. The study was approved by the ethics committee of the Medical Faculty of the University of Duisburg-Essen (19-8750-BO).

### 2.2 Statistical analyses

Continuous variables are presented as mean ± standard deviation (SD) for normally distributed and median with interquartile range (IQR) if skewed. Discrete variables are presented as counts and relative frequencies. Descriptive statistics were performed to assess the number of cases fulfilling criteria for and receiving ICP monitoring. Secondary descriptive analyses were performed to compare the initial presentation, intracranial injury patterns, and outcomes between cases with and without ICP monitoring. Statistical analyses were performed with Microsoft Office Excel 2016 (Microsoft Corporation, Redmont, WA, USA) and SAS Enterprise Guide 7.3 (SAS Institute, Cary, North Carolina, USA). Missing data was excluded from analysis.

## 3 Results

**Figure 2:**
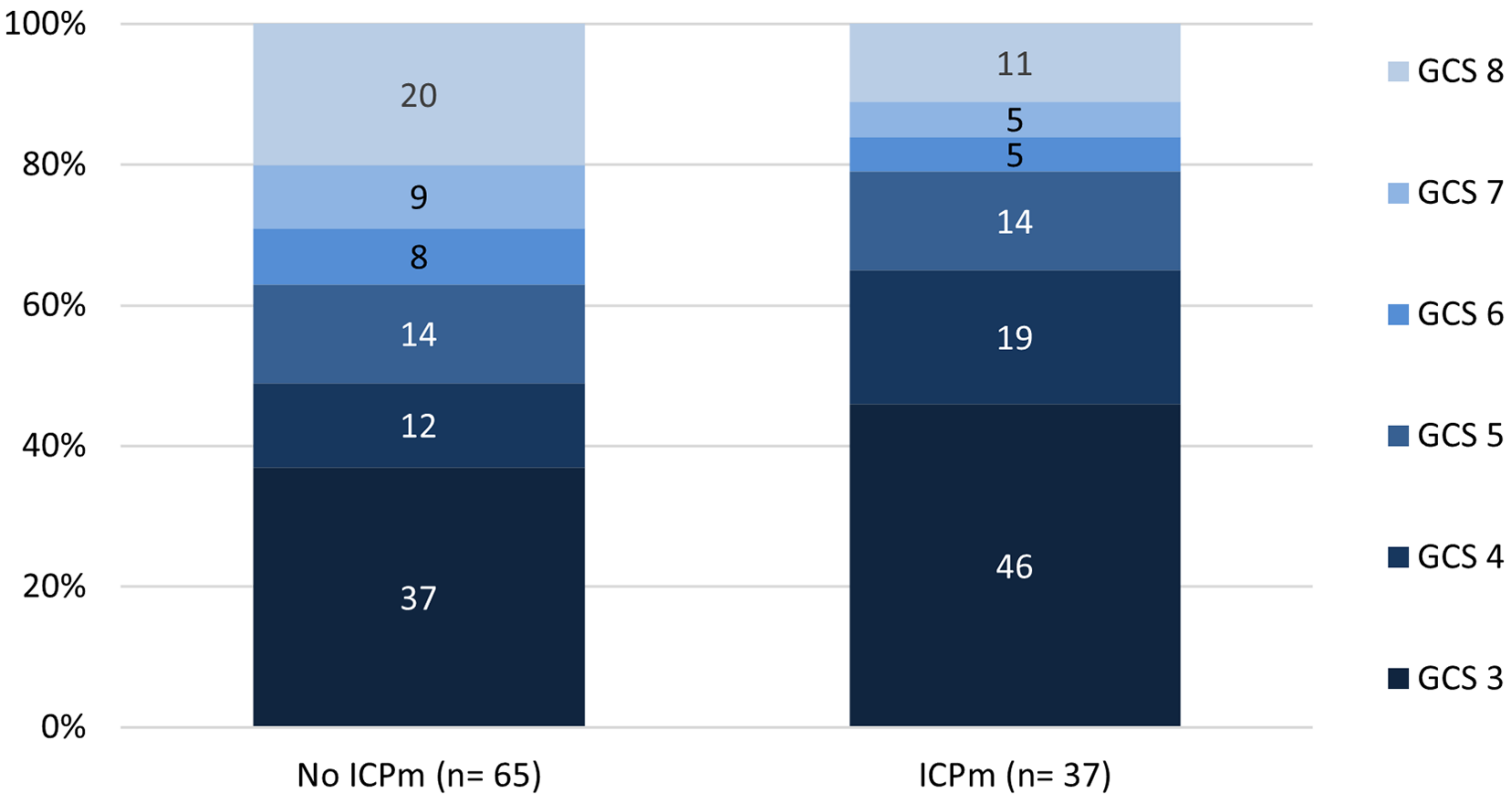
Initial Glasgow coma scale values grouped by ICP monitoring in children with severe traumatic brain injury. GCS, glascow-come-scale; ICPm, intracranial pressure monitoring.

During the study period, 217 cases <10 years with GCS ≤8 or neurosurgery due to TBI were reported to ESPED. One hundred two cases had an initial GCS ≤8, of which 37 (36%) received ICP monitoring (Table 1). In 15 of 37 (41%) cases, information on the type of inserted ICP monitor was provided (*n* = 11 (73%) parenchymal probe, *n* = 4 (27%) external ventricular drainage). Almost all included children had been previously healthy (91%). The monitored patients were older, had a more balanced gender distribution and lower admission temperature. Regarding admission weight and blood pressure no differences were observed. Of infants <1 year, only 27 (11%) received ICP monitoring (data not shown). Sixty-nine children (68%) were initially on invasive ventilation and 11 (11%) received vasopressors/inotropes in the emergency unit. In the group with ICP monitoring, the primary care was performed more frequently in the highest level trauma centers (Table 1). These patients had noticeably more often GCS levels ≤5 compared to patients without ICP monitoring (Figure 2).

**Table 1:** Baseline characteristics and outcomes of children <10 years with severe traumatic brain injury depending on invasive intracranial pressure monitoring.

	No ICP monitoring (*n* = 65)	ICP monitoring (*n* = 37)	Missing *n* (No ICP; ICP)
Age [years], median (IQR)	2.4 (0.4–4.8)	3.3 (1.8–5.8)	0; 0
Female, *n* (%)	25 (38)	15 (41)	0; 0
Height [cm], median (IQR)	88 (65–115)	108 (89–121)	28; 17
Weight [kg], median (IQR)	15 (7–20)	15 (13–20)	6; 2
Head circumference [cm], median (IQR)	45 (39–48)	50 (44–53)	41; 30
Mean arterial blood pressure [mmHg], mean ± SD	69 ± 22	69 ± 19	40; 22
Admission temperature [°C], mean ± SD	35.6 ± 2.3	34.9 ± 2.1	25; 18
Previous healthy, *n* (%)	59 (91)	34 (92)	0; 0
Primary care			
Highest level trauma center, *n* (%)	28 (56)	21 (72)	16; 8
PICU admission, *n* (%)	59 (95)	33 (92)	3; 1
GCS, median (IQR)	5 (3–7)	4 (3–5)	0; 0
Invasive ventilation, *n* (%)	38 (58)	31 (84)	0; 0
Vasopressors/inotropes, *n* (%)	5 (8)	6 (17)	5; 2
Both pupils fixed, *n* (%)	17 (32)	7 (23)	12; 7
Neuroimaging			
Cranial ultrasound, *n* (%)	13 (20)	4 (11)	0; 0
cCT, *n* (%)	53 (82)	37 (100)	0; 0
cMRI, *n* (%)	22 (34)	1 (3)	0; 0
Cerebral edema, *n* (%)	22 (37)	25 (68)	6; 0
Intracranial hemorrhage, (%)	56 (86)	33 (89)	0; 0
Epidural, *n* (%)	14 (22)	9 (24)	0; 0
Subdural, *n* (%)	36 (55)	18 (49)	0; 0
Subarachnoid, *n* (%)	18 (28)	21 (57)	0; 0
Parenchymal, *n* (%)	22 (34)	11 (30)	0; 0
Accompanying injuries, *n* (%)	54 (83)	33 (89)	0; 0
Eye, *n* (%)	26 (40)	12 (32)	0; 0
Heart, *n* (%)	10 (15)	5 (14)	0; 0
Thorax, *n* (%)	24 (37)	16 (43)	0; 0
Abdomen, *n* (%)	14 (22)	8 (22)	0; 0
Pelvic, *n* (%)	12 (18)	6 (16)	0; 0
Spine, *n* (%)	8 (12)	6 (16)	0; 0
Conservative treatment of intracranial hypertension			
Mannitol, *n* (%)	9 (15)	15 (41)	3; 0
Hypertonic saline, *n* (%)	0 (0)	8 (23)	5; 2
Steroids, *n* (%)	6 (10)	5 (14)	4; 1
Surgical treatment			
Hematoma clearance, *n* (%)	13 (20)	14 (38)	0; 0
Reposition of skull fracture, *n* (%)	5 (8)	3 (8)	0; 0
Decompressive craniectomy, *n* (%)	11 (18)	11 (31)	0; 0
Outcome			
Length of PICU stay, median (IQR)	8 (2–15)	7.5 (3–21)	15; 3
Mortality, *n* (%)	14 (22)	12 (32)	2; 0
Glasgow Outcome Scale at hospital discharge, median (IQR)	4 (0–5)	3 (0–4)	8; 3

### 3.1 Neuroimaging

Patients with sTBI received cranial computed tomography (cCT) in 88%. Cerebral edema was more frequent in monitored vs. non-monitored patients (68% vs. 37%), as well as subarachnoid hemorrhage (57% vs. 28%). The frequency of other intracranial injuries was largely similar between the groups, and accompanying injuries were reported in 85%.

### 3.2 Medical and surgical treatment

Mannitol to reduce intracranial hypertension was used in 24 patients (23%), hypertonic saline in 8 (8%), and steroids in 11 (10%). The ICP monitored group more often received conservative measures to reduce ICP (76% vs. 23%), as well as surgical therapies like clearance of hematoma or decompressive craniectomy (76% vs. 45%).

### 3.3 Outcomes

**Figure 3:**
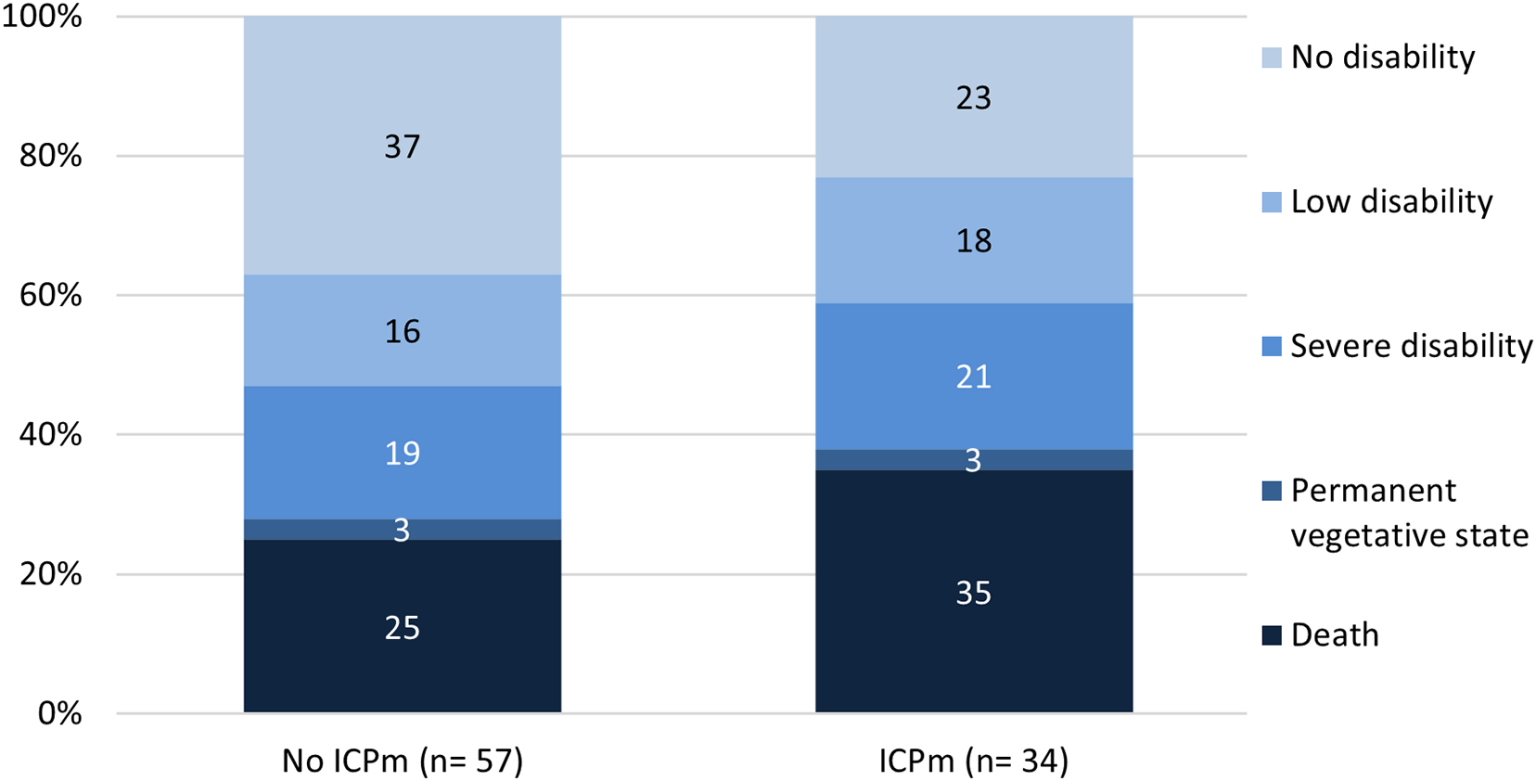
Glasgow outcome scale grouped by ICP monitoring in children with severe traumatic brain injury. GOS, Glasgow outcome scale, ICPm, intracranial pressure monitoring.

Seventy-four children (73%) survived until hospital discharge. Of 91 cases with reported outcome measured by Glasgow Outcome Scale (GOS) at hospital discharge, 32% survived without disabilities, 16% had mild disabilities, 20% severe disabilities and 3% were in a vegetative state. Of children with GCS ≤5, 37% died and 29% were in a vegetative state or suffered from severe disabilities. Overall, adverse outcomes were more frequent in the monitored group (Figure 3).

### 3.4 Coverage of the survey

No direct alternative data source was available to assess the completeness of the survey. Data published from the German nationwide hospital discharge data set (11) for the years 2014–2018 indicate that approximately 95 ICP probes (including extraventricular drains) are placed in children with TBI <10 years of age every year. Assuming that these numbers remained stable throughout the study period, the estimated coverage is 8.2%. No data source is available to estimate the number of TBI patients potentially eligible for ICP measurement.

## 4 Discussion

This study provides an overview of sTBI management in children <10 years between 2019 and 2022 in German children's hospitals. ICP monitoring was performed in approximately one third of cases. Mortality and adverse functional outcome were more frequent in the ICP monitored group, likely reflecting differences in baseline characteristics regarding patient age, intracranial pathologies, and treatment center.

ICP monitored children were older than non-monitored children. Particularly, infants below one year of age rarely received ICP monitoring, possibly due to the availability of transfontanellar ultrasound to monitor ventricular width, cerebral edema and perfusion. Of note, ICP monitored patients had lower initial GCS scores, presented with cerebral edema more often, were more often invasively ventilated, received vasopressors/inotropes and frequently suffered from multiple injuries. Thus, the indication to insert an ICP monitor seems to be driven by considerations beyond the state of consciousness, despite recommendations of current national and international guidelines (4, 6).

However, the performed ICP monitoring practice in Germany between 2019 and 2022 did not correspond to international sTBI guidelines that were in effect at that time. The German national TBI guideline did not clearly recommend ICP monitoring for patients with GCS ≤8, in contrast to the 2022 update (4, 8). This low monitoring rate is of interest, because it clearly deviates from the self-reported ICP monitoring rate of up to 86% in a nationwide survey (9). This reflects a gap between theoretical knowledge on the indication and the actual implementation.

Even though the guidelines have strong expert consensus regarding their recommendations on ICP monitoring in sTBI, the level of evidence underlying these recommendations is low (4, 6, 7) and it is still under debate whether ICP monitoring actually improves outcomes. While positive effects of diversion of cerebrospinal fluid on outcomes have been reported (12–14), other observational studies found no effect or even point in the opposite direction (15, 16). In the absence of sufficiently powered prospective randomized controlled trials, it remains unclear whether these findings truly reflect adverse effects of the interventions or the impossibility to completely adjust for confounding factors. The low level of evidence may cause uncertainty upon the decision whether to insert ICP monitoring or not, which may be driven by additional considerations, such as pending surgical or neurosurgical procedures and accompanying conditions.

It seems reasonable and has been reported in adults, that ICP monitored patient receive interventions to reduce intracranial pressure more often, both medical and surgical (15). This finding was also observed in our study and points towards important further considerations: ICP monitoring alone cannot improve patient outcomes, but has to be accompanied by prompt and adequate interventions to control intracranial hypertension (17). Further, potential complications such as infection, hemorrhage, mechanical disruption, malfunction and misplacement of the device have to be considered (18, 19). In clinical practice, non-invasive techniques to indirectly monitor ICP e.g., near-infrared spectroscopy, transcranial and transfontanellar Doppler ultrasound, fundoscopy and magnetic resonance imaging (MRI) are available to complement invasive ICP monitoring and may impact both the decision to insert ICP monitoring and the conservative management in the PICU.

This study is limited by the surveillance design, which covers only children's hospitals and relies on the compliance of participating centers. The small case number can be partially explained by the rarity of the condition, but it is evident that only a fraction of cases occurring during the study period were captured. With an estimated 8%, the completeness of this survey is at the lower range of literature reports (20) and lower than previously reported for ESPED surveys (21). Also, no data on treatment and device associated complications were available due to missing specific questions. No information was collected on the decision-making process of treating physicians to control for indication bias and information in the type of inserted ICP monitor was missing in more than half of cases.

## 5 Conclusion

ICP monitoring practice in severely head-injured children in Germany does not comply with current international guidelines. The reasons remain unclear but a contradictory expired national guideline may have hindered adherence to international guidelines. This guideline discrepancy is removed with the 2022 update of the German national TBI guideline, and a future study should elucidate whether subsequent changes in ICP monitoring practice occur. Educational interventions to promote the content of the updated guideline may further increase awareness of and compliance with current recommendations. For the future, randomized controlled studies to verify whether ICP monitoring in children with sTBI is beneficial and to untangle the effects of monitoring and ICP directed interventions to control ICP in monitored patients are indispensable.

## Data Availability

The raw data supporting the conclusions of this article will be made available by the authors, without undue reservation.
